# Relation of therapies for ankylosing spondylitis and psoriatic arthritis to risk of myocardial infarction: a nested case control study

**DOI:** 10.1186/s41927-021-00207-1

**Published:** 2021-07-29

**Authors:** Rachael Stovall, Christine Peloquin, David Felson, Tuhina Neogi, Maureen Dubreuil

**Affiliations:** 1 Boston University Medical Center, Boston, MA USA; 2 Section of Rheumatology, Boston University School of Medicine, 650 Albany Street, X-200, Boston, MA 02118 USA; 3 OptumLabs Visiting Scholar, OptumLabs, Eden Prairie, MN USA

**Keywords:** Ankylosing spondylitis, Psoriatic arthritis, Tumor necrosis factor inhibitors, Myocardial infarction

## Abstract

**Background:**

Risk of myocardial infarction (MI) is elevated in ankylosing spondylitis and psoriatic arthritis (AS/PsA) compared to the general population. We evaluated the risk of MI related to the use of tumor necrosis factor inhibitor (TNFi) and other therapies in AS/PsA.

**Methods:**

We conducted a nested case-control study using 1994–2018 data from OptumLabs® Data Warehouse, which includes de-identified medical and pharmacy claims, laboratory results, and enrollment records for commercial and Medicare Advantage enrollees. The database contains longitudinal health information on enrollees and patients, representing a diverse mixture of ages, ethnicities and geographical regions across the United States. Assessing AS/PsA separately, MI cases were matched to 4 controls by sex, age, diagnosis year and insurance type. We evaluated treatment within 6 months prior to MI including NSAIDs (AS referent), disease-modifying anti-rheumatic drug (DMARDs; PsA referent) and TNFi alone or in combinations. We evaluated the relation of treatment categories to MI risk using conditional logistical regression adjusting for confounders.

**Results:**

Among 26,648 AS subjects, there were 237 MI cases and 894 matched controls. Among 43,734 PsA subjects, there were 404 cases and 1596 controls. In AS, relative to NSAID use, the adjusted odds ratio (aOR) for MI among TNFi only users was 0.85 (95% CI 0.39–1.85) and for DMARD only users was 1.04 (95% CI 0.65–1.68). In PsA, relative to DMARD use, the aOR among TNFi only was 1.09 (95% CI 0.74–1.60). Combination therapies also had no effect.

**Conclusions:**

Among AS/PsA, no combination of therapies appeared to be protective or harmful with regards to MI. Future studies should capture more AS and PsA patients and include longer term follow up to further investigate this question.

**Supplementary Information:**

The online version contains supplementary material available at 10.1186/s41927-021-00207-1.

## Background

Individuals with ankylosing spondylitis (AS) and psoriatic arthritis (PsA) are at an increased risk of cardiovascular disease compared to the general population [1–4]. One meta-analysis estimates an odds ratio of 1.60 for myocardial infarction (MI) among those with AS [3] and another estimates 1.68 for PsA [5] compared to the general population. Several factors could explain this increased risk. First, AS and PsA are diseases with systemic inflammation which may accelerate atherosclerosis. Second, both are associated with an atherogenic lipid profile [6, 7]. Third, there are other specific cardiovascular abnormalities seen in these diseases. In AS individuals are at higher risk of aortic insufficiency, diastolic dysfunction and conduction disturbances [8–10]. In PsA there is a higher prevalence of aortic insufficiency and conduction disturbances, particularly atrioventricular block [11]. Both AS and PsA place individuals at higher risk of atrial fibrillation [11]. Fourth, non-steroidal anti-inflammatory drugs (NSAIDs) commonly used, especially in AS, could increase the risk of MI, as suggested by a large meta-analysis of subjects in the general and elderly population [12]. However, one large retrospective study of subjects with AS suggested NSAID use was associated with a decrease risk of vascular mortality among older adults [1]. This latter study did not assess for the effects of TNFi on vascular disease.

Pharmacological treatment of AS and PsA has changed in the past two decades and it is unclear how these medications could affect MI risk. To date, two different classes of drugs have dominated AS treatment, NSAIDs and tumor necrosis factor inhibitors (TNFi). The most recent treatment guidelines for AS recommend NSAIDs as first-line treatment reserving TNFi for those who have persistently high disease activity [13]. For both NSAIDs and TNFi, strong evidence demonstrates AS symptom control [13–15].

In treatment naïve patients with PsA, the latest guidelines recommend prescribing a TNFi first [16]. In the past, including 2015 guidelines, these patients would be started on a certain class of medications depending on their manifestations of PsA [17]. For example, NSAIDs were recommended as first line therapy for axial disease, enthesitis and dactylitis, while disease-modifying anti-rheumatic drugs (DMARDs) were recommended as first line treatment for peripheral arthritis and TNFi for nail involvement [17].

There are several mechanisms by which TNFi may reduce risk of MI in AS and PsA patients. Most directly, TNF inhibition may prevent both development and progression of atherosclerotic plaques through its anti-inflammatory effects on the endothelium [18]. Secondly, TNFi may reduce cardiovascular disease (CVD) risk through improvement in other CVD risk factors, including altered lipid profiles, a reduction in insulin resistance and the risk of diabetes, as has been found in patients with rheumatoid arthritis (RA) [19, 20] . Because AS commonly affects the spine and large joints, TNFi-induced disease control in AS may have an important benefit to exercise tolerance and physical activity, which would, in turn, improve CVD risk. The use of NSAIDs in AS has been associated with increased MI risk in certain situations [21–23]. Thus, any AS treatment strategy that is “NSAID sparing” may mitigate the MI risk conferred by NSAID use.

The existing literature surrounding the role of TNFi on MI in AS and PsA is minimal and inconsistent [4]. In RA, several systematic reviews have concluded that there is an overall protective effect of TNFi on risk of MI, despite some individual studies showing no effect [24–28]. Authors have speculated that differences in study design account for these discrepant results.

We sought to estimate the risk of MI in patients with AS and PsA, relative to the use of specific medication classes in US adults.

## Materials and methods

We conducted a nested case-control study using data from OptumLabs® Data Warehouse, which includes de-identified medical and pharmacy claims, laboratory results, and enrollment records for commercial and Medicare Advantage enrollees. The database contains longitudinal health information on enrollees and patients, representing a diverse mixture of ages, ethnicities and geographical regions across the US. Members in the database had full insurance coverage for physician, hospital, and prescription drug services [29].

### Study sample

**Fig. 1:**
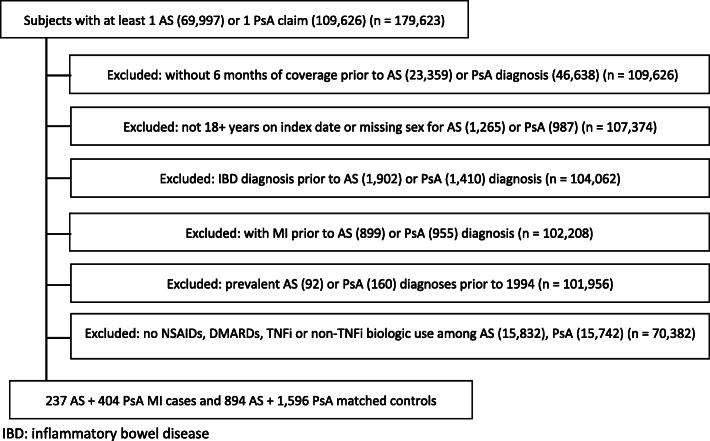
Selection Criteria

Claims were used to identify adults with AS and PsA ages 18 and older from 01/01/1994 to 12/31/2018. We chose to start the study in 1994 in order to maximize our study sample. Subjects were required to have medical and pharmacy coverage for at least 6 months prior to their first AS or PsA claim in the database (which may or may not coincide with their first diagnosis of AS/PsA) and to have filled a prescription for at least one medication from categories of NSAID, DMARD or TNFi. AS and PsA subjects were eligible for inclusion on the date of their first AS or PsA claim. If a subject had both AS and PsA diagnoses, we categorized them by the first diagnosis listed. MI was defined by ICD 9/10 diagnostic codes (Supplemental Table 1), and the date of first MI during the study period was defined as the index date, after which subjects no longer contributed data for this case-control study. We excluded subjects who previously had an MI, subjects of unknown sex, and subjects with inflammatory bowel disease any time prior to AS or PsA diagnosis (Fig. 1). The latter were excluded because they may have different treatment patterns than AS and PsA subjects.

### Study design

The outcome of interest was MI as defined by diagnostic codes. We evaluated subjects with AS and PsA separately. For each MI case, we selected up to 4 controls without an MI, matching to each case by sex, age (+/− 3 years), year of AS/PsA diagnosis (+/− 1 year) and insurance type (Medicare Advantage or commercial insurance). Subjects were matched using risk-set sampling. Thus, each subject could be included as a control before they had an MI. The index date for MI cases was the date of the MI claim, and for controls, index date was the date of the MI claim for the matched case.

### Exposure assessment

The exposure of interest was AS/PsA drug treatment within 6 months prior to the index date, based on prescription claims data. A prescription claim indicates that a medication was filled, as the insurance company was billed at that time. Due to the nature of claims data, we were unable to measure the duration of these treatments, and instead categorized their use as present or absent during our exposure assessment period. We considered treatment categories of: NSAID only, DMARD only, TNFi only, and all combinations of these categories. Janus kinase inhibitors and non-TNFi biologics were not included in this study as there were an insufficient number of subjects on these medications. In our main analysis, we classified subjects who were initially on an NSAID only and then switched to a TNFi only during the exposure assessment period in our TNFi only category. We then performed a sensitivity analysis in which we considered those subjects both: (i) in a separate exposure category and (ii) combined with other subjects who had both NSAID and TNFi use. The purpose of this sensitivity analysis was to most accurately assess for any differences among those exposed to TNFi alone versus TNFi in combination with NSAIDs.

### Covariate assessment

Potential confounders for the relationship between treatment category and MI outcome were assessed during the period prior to study follow-up. Age, sex and insurance type were determined from administrative data. Claims codes were used to define the presence of chronic kidney disease (CKD), diabetes, hypertension, ischemic heart disease, liver disease, peptic ulcer disease, psoriasis, RA, obesity (defined as body mass index > 30 kg/m^2^), statin use, and smoking status. We assessed for psoriasis because it is an independent risk factor for cardiovascular disease [30]. Subjects with a prior diagnosis of RA were included because it is not uncommon for patients to initially have a diagnosis of RA prior to receiving a diagnosis of AS or PsA. In a previous validation study by our group, the absence of an RA code improved the positive predictive value by less than 1% (71.8% for a single AS code and 72.5% for a single AS code and absence of an RA code) [31]. Similarly, in previous studies validating a PsA diagnosis, the exclusion of an RA diagnosis did not change the positive predictive value (85.1% allowing for RA codes, and 86.1% excluding RA codes) [32]. We also adjusted for a record of erythrocyte sedimentation rate (ESR) or c-reactive protein (CRP) having been performed within 1 year prior to an AS/PsA diagnosis as well as at least 1 rheumatology visit in the year prior to an AS/PsA diagnosis (versus no visits). These variables were used as surrogates for disease activity, as they are more likely to be present among subjects with high disease activity, and there is no validated disease activity measure available in these data. We reviewed 100 random case profiles to validate our outcome (MI) to ensure that code selection and programming was appropriate.

### Statistical analysis

Evaluating AS and PsA separately, the odds of MI were assessed in each treatment exposure category (NSAID, DMARD and TNFi) with adjustment for potential confounders using conditional logistic regression. All combinations of treatment categories were assessed. For AS, NSAID use only was the referent group as NSAIDs are considered first line therapy for this disease [13]. For PsA, DMARD use only was the referent group given its historical use as first line therapy [16]. Then we performed an analysis of AS and PsA combined with NSAID use as the referent group. The purpose of this was to analyze the largest number of subjects over the greatest amount of time to maximize power to detect any cardioprotective (or harmful) effects of these medical therapies. We performed several sensitivity analyses including: matching cases and controls based on history of ischemic heart disease, adjusting for aspirin use as a covariate in the model, creating alternative drug exposure categorization to include subjects who switched from DMARD to TNFi within the TNFi category, and examining alternative (3- and 14- month) exposure assessment periods. Lastly, we performed a conditional logistic regression model analysis considering each drug category as a binary variable and assessed the effect of each category after adjustment for other confounders.

### Ethical approval

Since this study involved analysis of pre-existing, de-identified data, it was exempt from Institutional Review Board approval.

## Results

### Ankylosing spondylitis

There were 26,648 adults with AS who met inclusion criteria for this study. Table 1 shows the baseline characteristics of the subjects. Among AS matched subjects, the mean age was 62.8 (SD +/− 11.1) years and 45.6% were women. There were 237 MI cases and 894 matched controls with AS. There was a trend toward a greater prevalence of traditional MI risk factors among MI cases, including diabetes, smoking and a diagnosis of ischemic heart disease (Table 1). Table 2 shows the odds of MI per each drug category. In AS, relative to NSAID use, the OR for MI among TNFi only users was 0.85 (95% CI 0.39–1.85) and for DMARD only users was 1.04 (95% CI 0.65–1.68).

**Table 1:** Characteristics of Cases and Controls

	AS		*P*-value	PsA		*P*-value
	MI Cases	Controls		MI Cases	Controls	
Subjects, n	237	894		404	1596	
Female, n (%)	110 (46.4%)	406 (45.4%)	0.7836	167 (41.3%)	660 (41.4%)	0.9951
Age, mean +/− SD	63.0 +/− 11.3	62.8 +/− 11.0	0.7100	61.5 + − 11.2	61.4 + − 11.2	0.8300
Chronic kidney disease, n (%)	13 (5.5%)	36 (4.0%)	0.3269	22 (5.4%)	45 (2.8%)	0.0088
Diabetes, n (%)	62 (26.2%)	191 (21.4%)	0.1152	138 (34.2%)	329 (20.6%)	< 0.0001
Ischemic heart disease, n (%)	66 (27.8%)	131 (14.7%)	< 0.0001	100 (24.8%)	209 (13.1%)	< 0.0001
Liver disease, n (%)	< 11^a^ (< 5.0%)	22 (2.5%)	NS^a^	20 (5.0%)	58 (3.6%)	0.2221
Peptic ulcer disease, n (%)	58 (24.5%)	233 (26.1%)	0.6186	71 (17.6%)	297 (18.6%)	0.6316
Psoriasis, n (%)	42 (17.7%)	173 (19.4%)	0.5697	259 (64.1%)	1073 (67.2%)	0.2347
Rheumatoid arthritis, n (%)	76 (32.1%)	237 (26.5%)	0.0891	99 (24.5%)	340 (21.3%)	0.1649
Body mass index > 30 kg/m^2^), n (%)	33 (13.9%)	104 (11.6%)	0.3365	75 (18.6%)	201 (12.6%)	0.0019
Statin use, n (%)	95 (40.1%)	308 (34.5%)	0.1075	147 (36.4%)	565 (35.4%)	0.7118
Smoking status, n (%)	28 (11.8%)	54 (6.0%)	0.0023	41 (10.1%)	76 (4.8%)	< 0.0001
Rheumatology visit in year prior to diagnosis, n (%)	103 (43.5%)	406 (45.4%)	0.8200	214 (53.0%)	911 (57.1%)	0.0200
ESR/CRP ordered in year prior to diagnosis, n (%)	95 (40.1%)	349 (39.0%)	0.7693	175 (43.3%)	651 (40.8%)	0.3567

*P* values were labeled as non-significant, or NS, when p was > 0.05 and was unable to be reported due to the risk of calculating small numbers of subjects (< 11)

*SD* Standard deviation, *ESR* Erythrocyte sedimentation rate, *CRP* c-reactive protein, *NS* Non-significant

^a^Counts under 11 subjects were not reported to prevent inadvertent subject identification

**Table 2:** Odds of Drug Treatment Categories within 6 Months of MI Among Adults with AS and PsA separately and combined

	Ankylosing Spondylitis		Psoriatic Arthritis		Combined	
	Cases/Controls	Fully Adjusted OR^b^ (95% CI)	Cases/Controls	Fully Adjusted OR^b^ (95% CI)	Cases/Controls	Fully Adjusted OR^b^ (95% CI)
NSAID use only	135/536	1.0 (ref)	106/430	0.84 (0.61, 1.17)	241/966	1.0 (ref)
DMARD use only	37/141	1.04 (0.65, 1.68)	112/432	1.0 (ref)	149/573	1.13 (0.87, 1.48)
TNFi use only	< 11^a^/49	0.85 (0.39, 1.85)	> 65^a^/244	1.09 (0.74, 1.60)	76/293	1.19 (0.85, 1.67)
NSAID, DMARD	27/86	1.29 (0.76, 2.20)	62/248	0.97 (0.67, 1.40)	89/334	1.17 (0.86, 1.58)
NSAID, TNFi	11/29	1.77 (0.79, 3.98)	18/91	0.83 (0.47, 1.48)	29/120	1.15 (0.72, 1.83)
DMARD, TNFi	< 11^a^/34	0.91 (0.38, 2.16)	> 20^a^/96	0.77 (0.45, 1.34)	31/130	0.93 (0.59, 1.47)
NSAID, DMARD, TNFi	< 11^a^/19	1.99 (0.78, 5.12)	> 15^a^/55	1.23 (0.67, 2.25)	26/74	1.55 (0.93, 2.57)

*OR* Odds ratio, *NSAID* Nonsteroidal anti-inflammatory drug, *TNFi* Tumor necrosis factor inhibitor, *DMARD* Symptom modifying anti-rheumatic drug

^a^Counts under 11 subjects were not reported to prevent inadvertent subject identification. Additional counts masked to protect back-calculation of these small numbers

^b^Adjusted for age, chronic kidney disease, diabetes, hypertension, ischemic heart disease, liver disease, peptic ulcer disease, psoriasis, rheumatoid arthritis, obesity, statin use, smoking status, rheumatology visits in the year prior to study eligibility, and erythrocyte sedimentation rate (ESR) or c-reactive protein (CRP) ordered in the year prior to study eligibility

### Psoriatic arthritis

There were 43,734 adults with PsA who met inclusion criteria for this study. Baseline characteristics among PsA, as listed in Table 1, show the mean age was 61.4 (SD +/− 11.2) years and 41.4% were women. There were 404 MI cases and 1596 matched controls with PsA. In PsA, relative to DMARD use, the aOR with TNFi only exposure was 1.09 (95% CI 0.74–1.60).

### Ankylosing spondylitis and psoriatic arthritis combined

Among AS and PsA, after adjustment for potential confounders, there were no combinations of NSAID, DMARD and TNFi use that resulted in a significantly increased or decreased OR for MI. Table 2 also shows our combined analysis of AS and PsA subjects using NSAID as the referent group. We found no association between any of the medication categories and risk of MI.

### Sensitivity analyses

In our regression model assessing NSAIDs, DMARDs and TNFi as binary variables, after adjustment for other confounders, we found no association for the effects of any drug category on MI, relative to non-use. In AS the aOR for NSAID use was 1.45 (95% CI 0.92–2.29), for DMARD use was 1.35 (95% CI 0.88–2.07) and for TNFi use was 1.27 (95% CI 0.79–2.05). In PsA, the aOR for NSAID use was 0.97 (95% CI 0.74–1.27), for DMARD use was 1.08 (95% CI 0.82–1.42) and for TNFi use was 1.07 (95% CI 0.79–1.45).

All of our other sensitivity analyses also yielded null results (data not shown). Specifically, we found no difference in risk of MI with regards to matching cases and controls based on a history of ischemic heart disease, nor with adjustment for aspirin use. The results were unchanged when performing alternative drug exposure categorization with subjects who switched from a DMARD to a TNFi included within the TNFi category. Similarly, results were unchanged using 3- and 14- month exposure assessment periods relative to the 6 month period in the primary analysis.

Supplemental Table 2 shows the results of a univariate analysis for the confounders of this study in a logistic regression model assessing AS and PsA subjects combined. The following were associated with a higher risk of MI: diabetes (aOR 1.63, 95% CI 1.31–2.04), hypertension (aOR 1.34, 95% CI 1.08–1.67), ischemic heart disease (aOR 2.17, 95% CI 1.70–2.76) and smoking status (aOR 2.24, 95% CI 1.61–3.12).

Supplemental Table 3 of this study lists the prescriptions filled by AS and PsA subjects including specific NSAIDs, DMARDs and TNFi. The most common NSAIDs prescribed were meloxicam (21.1%), celecoxib (17.3%), diclofenac (14.4%), naproxen (10.9%) and ibuprofen (9.5%). The most common DMARDs were methotrexate (56.6%), hydroxychloroquine (14.3%), sulfasalazine (8%), leflunomide (7%) and apremilast (5.3%). Lastly, the most common TNFi were adalimumab (44.5%), etanercept (38.2%), infliximab (12.2%), golimumab (2.7%) and certolizumab (2.3%).

## Discussion

This large US-based study is one of the first to examine the risk of MI in AS and PsA relative to treatment. We report the absence of any cardioprotective benefit from or increased risk with TNFi treatment after adjusting for traditional cardiovascular risk factors. While some studies have shown that TNFi appear protective against MI in RA [28], our findings do not support such an effect in AS or PsA.

There are potential explanations for our study’s findings. AS/PsA patients do not have as high of a risk for CVD when compared to RA patients. This could explain why TNFi do not appear to reduce the risk of MI in AS patients [2]. While a majority of studies show that PsA is associated with an increased risk of MI, this is not consistent across all studies [4]. It may be that patients with AS/PsA receive TNFi later in their disease course, or at an older age than RA patients, and therefore do not experience the potential CVD benefits as irreversible inflammation and plaque deposition have already taken place. This is because axial spondyloarthritis can take 5–10 years to be diagnosed from symptom onset [33, 34], whereas RA is often diagnosed on average 3–4 months after symptoms begin [35], though these estimates vary over time and by country.

It is difficult to compare our results due to the paucity of literature on this topic. Ogdie et al. retrospectively analyzed a longitudinal UK cohort of patients with PsA, psoriasis, and RA finding that all three groups had a higher risk of MI compared to the general population [36]. This study combined DMARD and TNFi use into one group and controlled for NSAID use. They found that these subjects, whether taking DMARD/TNFi or not, all had an elevated risk of MI. Notably, those with PsA, whether on DMARD/TNFi or not, had a very similar hazards ratio (HR) (1.36, 95% confidence interval (CI) 1.01–1.84 and 1.36, 95% CI 1.04–1.77 respectively). We are therefore unable to tell from this study if there are certain medical therapies that impact the risk of MI in PsA.

Sparks et al. analyzed patients with PsA, psoriasis and RA who already had an initial cardiovascular event (CVE) (MI, stroke or coronary revascularization procedure) and assessed for the risk of a subsequent CVE relative to medical therapies. They found that categories of DMARD, TNFi and non-biologic TNFi did not significantly impact the risk of a subsequent CVE [37]. NSAID use was not assessed, nor was MI analyzed separately from other CVE. These findings cannot be directly compared to our results as we purposefully excluded patients who had a prior MI.

Haroon et al. performed a large retrospective cohort study in Ontario, Canada and found among adults with AS aged 66 years and older, NSAID use was associated with a reduced risk of vascular mortality (HR 0.1, 95% CI, 0.01–0.61; *P* = 0.01) [1]. It is unclear if this is due to its effect on AS disease activity leading to less CVD, or if patients taking NSAIDs were healthier at baseline. NSAIDs are generally prescribed with caution in older adults. While the study controlled for comorbidities such as hypertension, diabetes, coronary artery disease, and CKD it did not control for hyperlipidemia, obesity, tobacco use, diet or physical activity.

### Strengths and limitations

This study has several strengths and limitations worth noting. First, our use of a large US claims data set allowed us to identify a large population of AS/PsA subjects, and therefore greater power to assess for an effect of treatment category on MI risk over smaller data sets. Second, we included only subjects who used at least one medication for AS/PsA treatment, thereby reducing confounding by indication; AS/PsA subjects whose disease was mild enough that they did not require a prescription medication were not included. We felt confident that those on DMARDs or TNFi had an inflammatory disease. However, this study is limited by the potential for misclassification in that over-the-counter NSAIDs could not be assessed. This misclassification is expected to bias the results for the non-NSAID exposure categories toward the null, (e.g.- making subjects categorized as “TNFi only” users appear more similar to the referent “NSAID user” group) than if the exposure classification was perfect. Next, we had a small sample size of subjects with AS on TNFi alone, which resulted in insufficient power, and therefore results should therefore be interpreted with great caution. Additionally, we could not account for confounding according to AS/PsA disease activity or functional status, as these variables were not available in the claims data set used for this study. In the absence of disease activity measures, we adjusted for rheumatology appointments and inflammatory marker testing as proxies for disease severity. However, people with more active or severe AS/PsA may be more likely to be prescribed TNFi and may have an increased risk of MI on the basis of AS/PsA activity/severity, rather than based on the treatment. Because of the nature of private health insurance in the US, in which many people have a change or disruption in insurer coverage due to a change in their/their spouse’s employment, it was not possible to assess the effect of long-term drug use. Next, diagnostic codes for MI have not been validated to our knowledge. However, our internal validation study confirmed the presence of MI in all 100 randomly selected cases. Lastly, no data on other cardiovascular events such as stroke or death due to CVD was investigated in this study.

Our study did not have sufficient power to detect differences in MI risk among those on various NSAIDs. We recently published a study on the risk of MI in AS using data from a large UK primary care-based data set, demonstrating that diclofenac (NSAID) is associated with MI risk in AS, while naproxen (NSAID) is not [23]. In the UK, biologic medications may be used in a different AS patient subset, and may be prescribed by consultants; therefore, prescriptions for TNFi in the primary-care data were insufficient to assess for an effect of TNFi on MI.

## Conclusions

In this large sample of US adults with AS and PsA, no combination of NSAIDs, DMARDs or TNFi therapy appeared protective against MI. Future studies should capture more AS and PsA patients and include longer term follow up to further investigate this question.

## Supplementary Information

**Additional file 1: Supplemental Table 1.** List of ICD 9/10 Codes Used to Identify Myocardial Infarction. **Supplemental Table 2.** Univariate Analysis Results for Confounders in a Logistic Regression Model: Analysis of AS and PsA Subjects Combined. **Supplemental Table 3.** List of Therapies and Frequency of Their Use During the Study Period.

## Data Availability

The datasets generated and/or analyzed during the current study are not publicly available due to restrictions from OptumLabs®. The data use requires researchers to pay for rights to use and access the data. Any and all code that the authors of this manuscript wrote can be exported via secure file transfer protocol to ensure it does not include any patient level information and be shared. This is available from the corresponding author on reasonable request.

## Abbreviations

aOR: Adjusted odds ratio
AS: Ankylosing spondylitis
CI: Confidence interval
CKD: Chronic kidney disease
CVD: Cardiovascular disease
CRP: C-reactive protein
CVE: Cardiovascular event
DMARD: Disease-modifying anti-rheumatic drug
ESR: Erythrocyte sedimentation rate
HR: Hazards ratio
MI: Myocardial infarction
NSAID: Non-steroidal anti-inflammatory drug
PsA: Psoriatic arthritis
RA: Rheumatoid arthritis
TNFi: Tumor necrosis factor inhibitor
