# Supplementary material for: Relation of therapies for ankylosing spondylitis and psoriatic arthritis to risk of myocardial infarction: a nested case control study
Source: BMC Rheumatol. 2021 Jul 29;5:36. doi: 10.1186/s41927-021-00207-1 (PMC8320220; doi:10.1186/s41927-021-00207-1)
Supplement: Supplementary file 1 — Additional file 1: Supplemental Table 1. List of ICD 9/10 Codes Used to Identify Myocardial Infarction. Supplemental Table 2. Univariate Analysis Results for Confounders in a Logistic Regression Model: Analysis of AS and PsA Subjects Combined. Supplemental Table 3. List of Therapies and Frequency of Their Use During the Study Period. [file 41927_2021_207_MOESM1_ESM.docx]

**Appendix and Data Supplements**

**Supplemental Table 1.** List of ICD 9/10 Codes Used to Identify Myocardial Infarction

| **Coding system** | **Code** | **Description** |
| --- | --- | --- |
| ICD-9-CM | 410 | Acute myocardial infarction |
| ICD-9-CM | 410.0 | Acute myocardial infarction of anterolateral wall |
| ICD-9-CM | 410.01 | Acute myocardial infarction of anterolateral wall, initial episode of care |
| ICD-9-CM | 410.1 | Acute myocardial infarction of other anterior wall |
| ICD-9-CM | 410.11 | Acute myocardial infarction of other anterior wall, initial episode of care |
| ICD-9-CM | 410.2 | Acute myocardial infarction of inferolateral wall |
| ICD-9-CM | 410.21 | Acute myocardial infarction of inferolateral wall, initial episode of care |
| ICD-9-CM | 410.3 | Acute myocardial infarction of inferoposterior wall |
| ICD-9-CM | 410.31 | Acute myocardial infarction of inferoposterior wall, initial episode of care |
| ICD-9-CM | 410.4 | Acute myocardial infarction of other inferior wall |
| ICD-9-CM | 410.41 | Acute myocardial infarction of other inferior wall, initial episode of care |
| ICD-9-CM | 410.5 | Acute myocardial infarction of other lateral wall |
| ICD-9-CM | 410.51 | Acute myocardial infarction of other lateral wall, initial episode of care |
| ICD-9-CM | 410.6 | True posterior wall infarction |
| ICD-9-CM | 410.61 | True posterior wall infarction, initial episode of care |
| ICD-9-CM | 410.7 | Subendocardial infarction |
| ICD-9-CM | 410.71 | Subendocardial infarction, initial episode of care |
| ICD-9-CM | 410.8 | Acute myocardial infarction of other specified sites |
| ICD-9-CM | 410.81 | Acute myocardial infarction of other specified sites, initial episode of care |
| ICD-9-CM | 410.9 | Acute myocardial infarction of unspecified site |
| ICD-9-CM | 410.91 | Acute myocardial infarction of unspecified site, initial episode of care |
| ICD-10-CM | I21 | Acute myocardial infarction |
| ICD-10-CM | I21.0 | ST elevation myocardial infarction of anterior wall |
| ICD-10-CM | I21.01 | ST elevation myocardial infarction involving left main coronary artery |
| ICD-10-CM | I21.02 | ST elevation myocardial infarction involving left anterior descending coronary artery |
| ICD-10-CM | I21.09 | ST elevation myocardial infarction involving other coronary artery of anterior wall |
| ICD-10-CM | I21.1 | ST elevation myocardial infarction of inferior wall |
| ICD-10-CM | I21.11 | ST elevation myocardial infarction involving right coronary artery |
| ICD-10-CM | I21.19 | ST elevation myocardial infarction involving other coronary artery of inferior wall |
| ICD-10-CM | I21.2 | ST elevation myocardial infarction involving other sites |
| ICD-10-CM | I21.21 | ST elevation myocardial infarction involving left circumflex coronary artery |
| ICD-10-CM | I21.29 | ST elevation myocardial infarction involving other sites |
| ICD-10-CM | I21.3 | ST elevation myocardial infarction of unspecified site |
| ICD-10-CM | I21.4 | Non-ST elevation myocardial infarction |
| ICD-10-CM | I21.9 | Acute myocardial infarction, unspecified |
| ICD-10-CM | I21.A9 | Other myocardial infarction type |

**Supplemental Table 2.** Univariate Analysis Results for Confounders in a Logistic Regression Model: Analysis of AS and PsA Subjects Combined

| Adjusted OR^+^  (95% CI) | |
| --- | --- |
| NSAID use only | 1.0 (ref) |
| SMARD use only | 1.13 (0.87-1.48) |
| TNFi use only | 1.19 (0.85-1.67) |
| NSAID, SMARD | 1.17 (0.86-1.58) |
| NSAID, TNFi | 1.15 (0.72-1.83) |
| SMARD, TNFi | 0.93 (0.59-1.47) |
| NSAID, SMARD, TNFi | 1.55 (0.93-2.57) |
|  |  |
| Age | 1.06 (1.00-1.13) |
| Chronic kidney disease | 1.31 (0.85-2.03) |
| ESR/CRP in year prior | 1.08 (0.87-1.34) |
| Diabetes | 1.63 (1.31-2.04) |
| Hypertension | 1.34 (1.08-1.67) |
| Ischemic heart disease | 2.17 (1.70-2.76) |
| Liver disease | 1.15 (0.72-1.85) |
| Obesity | 1.24 (0.95-1.62) |
| Psoriasis | 0.82 (0.66-1.02) |
| Peptic ulcer disease | 0.78 (0.62-0.98) |
| Rheumatoid arthritis | 1.24 (0.98-1.58) |
| Rheumatology visits (Y/N) | 1.24 (0.97-1.58) |
| Smoking status | 2.24 (1.61-3.12) |
| Statin use | 0.82 (0.66-1.01) |

+Adjusted for age, chronic kidney disease, diabetes, hypertension, ischemic heart disease, liver disease, peptic ulcer disease, psoriasis, rheumatoid arthritis, obesity, statin use, smoking status, rheumatology visits in the year prior to study eligibility, and erythrocyte sedimentation rate (ESR) or c-reactive protein (CRP) ordered in the year prior to study eligibility

**Supplemental Table 3.** List of Therapies and Frequency of Their Use During the Study Period

| **Drug class** | **Medication** |
| --- | --- |
| **NSAID (%)** | celecoxib (17.3), diclofenac (14.4), diflunisal (0.6), etodolac (3.0), fenoprofen (<0.4), flurbiprofen (0.4), ibuprofen (9.5), indomethacin (5.0), ketoprofen (0.9), ketorolac (1.5), meclofenamate (<0.4), meloxicam (21.1), nabumetone (4.6), naproxen (10.9), oxaprozin (1.4), piroxicam (1.9), rofecoxib (3.7), salsalate (0.6), sulindac (1.5), tolmetin (<0.4), trisalicylate (<0.4) and valdecoxib (1.1) |
| **SMARD (%)** | apremilast (5.3), auranofin (<0.4), azathioprine (1.7), baricitinib (0), chloroquine (<0.4), cyclophosphamide (<0.4), cyclosporine (3.9), hydroxychloroquine (14.3), leflunomide (7.0), methotrexate (56.6), mycophenolate (1.8), sulfasalazine (8.0), thiomalate (<0.4), and tofacitinib (0.8) |
| **TNFi (%)** | adalimumab (44.5), certolizumab (2.3), etanercept (38.2), golimumab (2.7) and infliximab (12.2) |
| **Non-TNF biologics^*^ (%)** | abatacept (30.3), anakinra (<0.4), guselkumab (0), ixekizumab (1.7), sarilumab (0), secukinumab (40.2), tocilizumab (3.9), and ustekinumab (23.6) |
| * not included in present analysis | |
